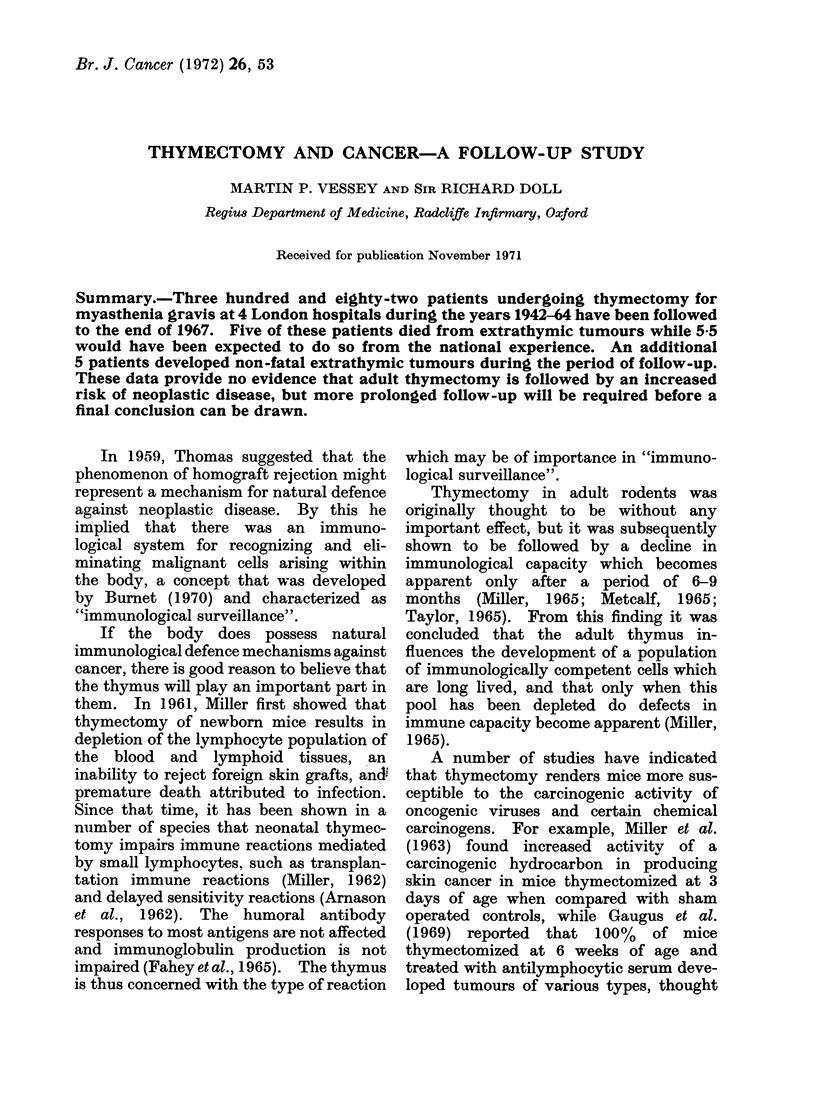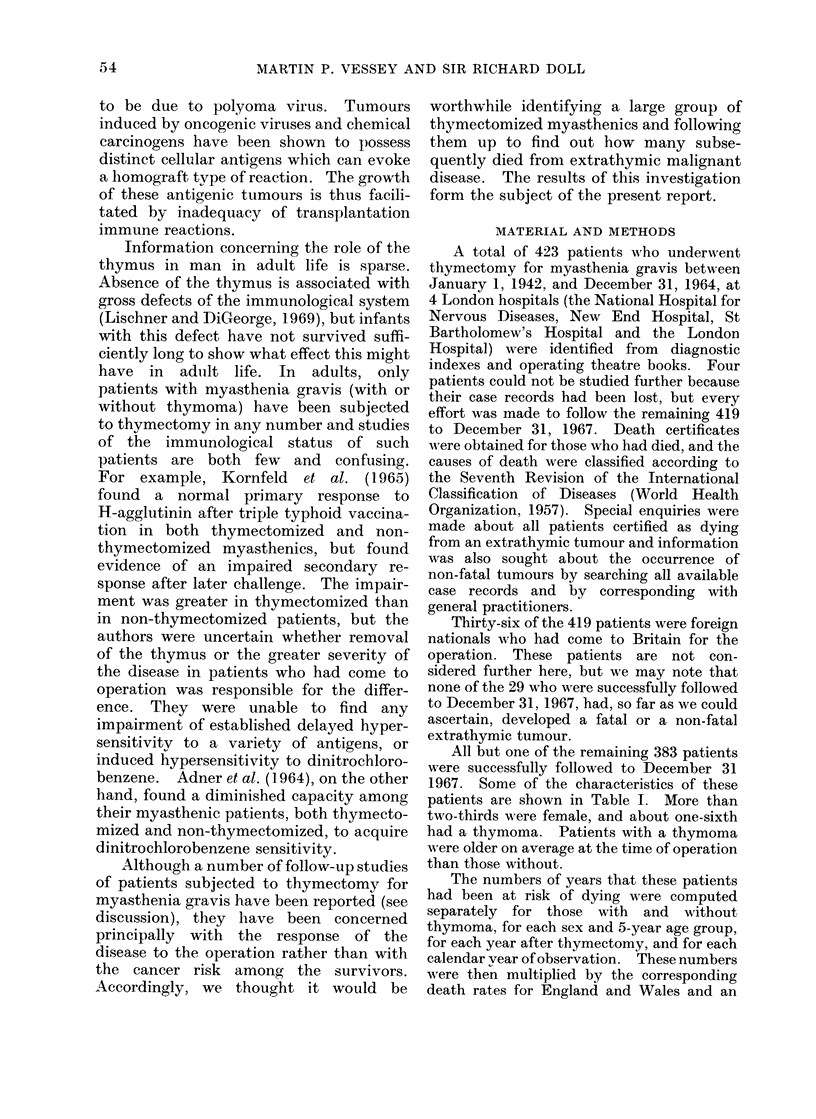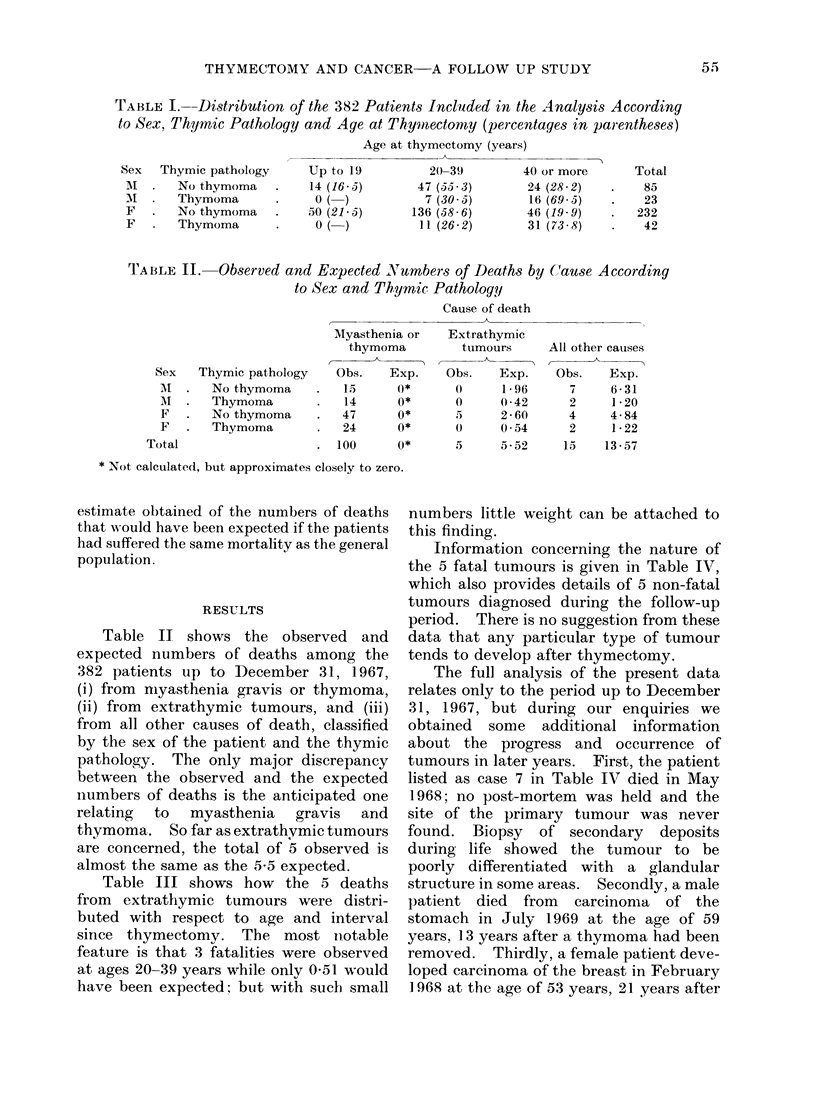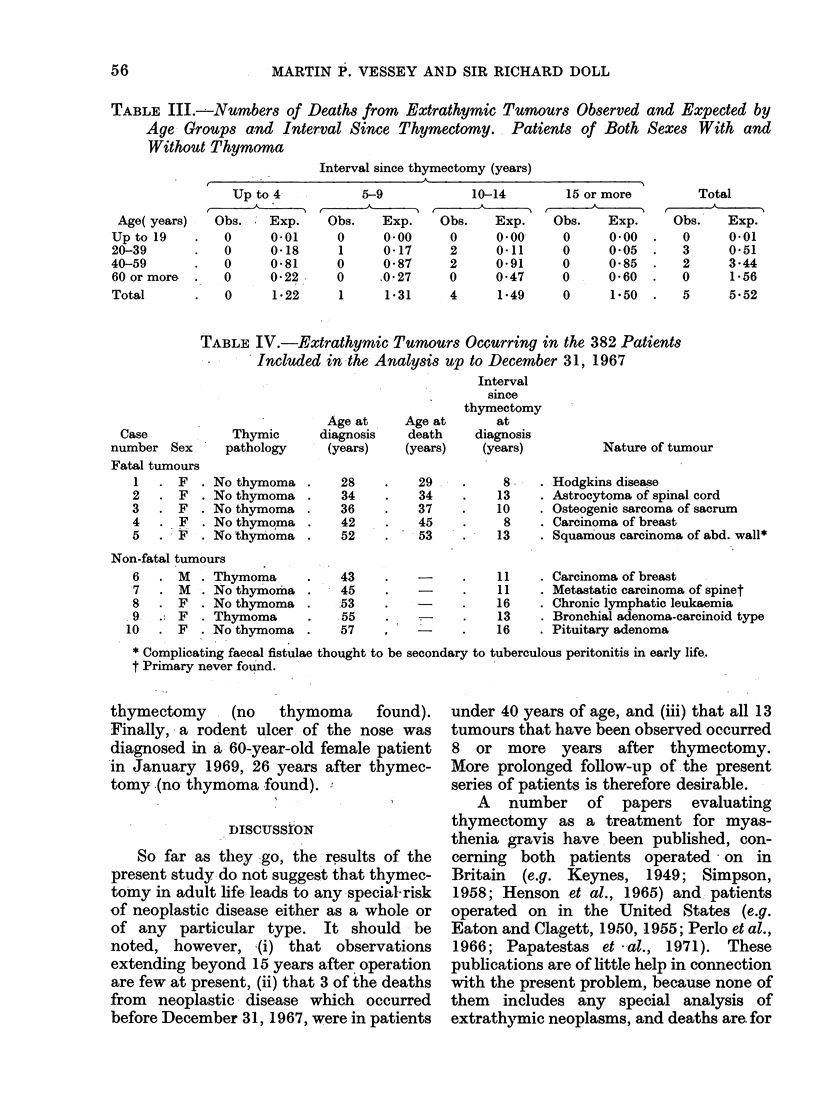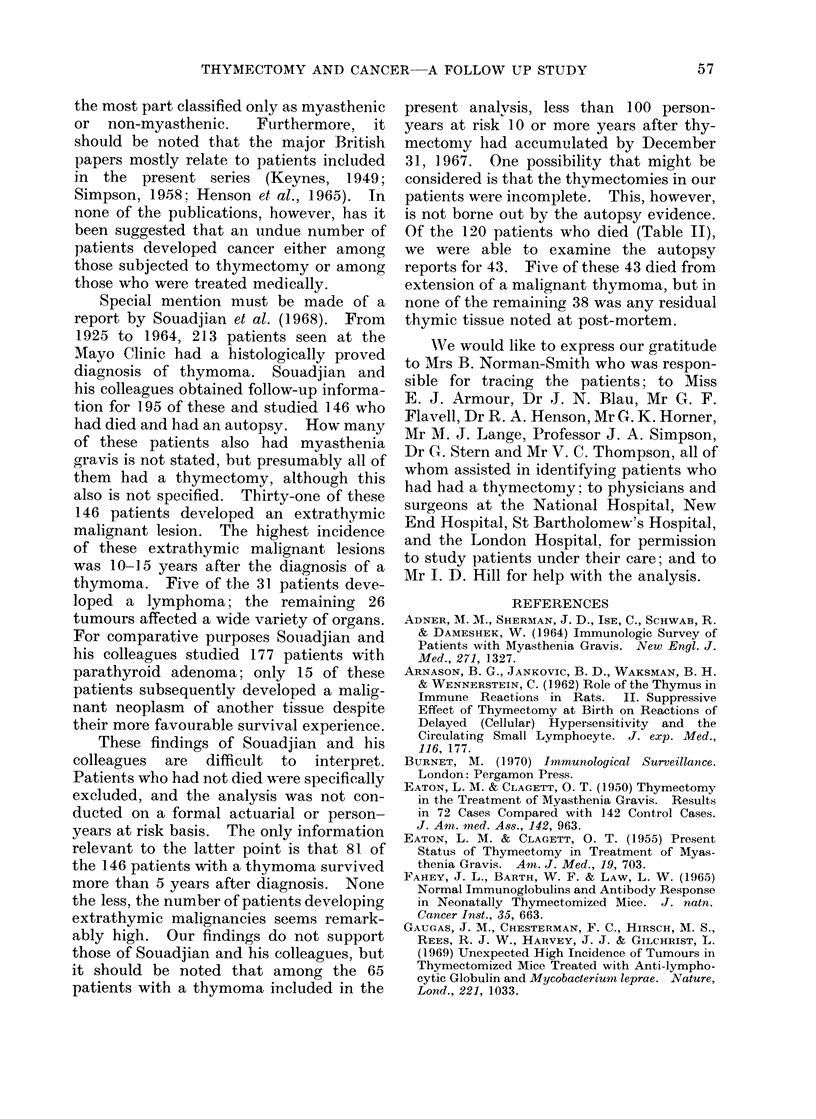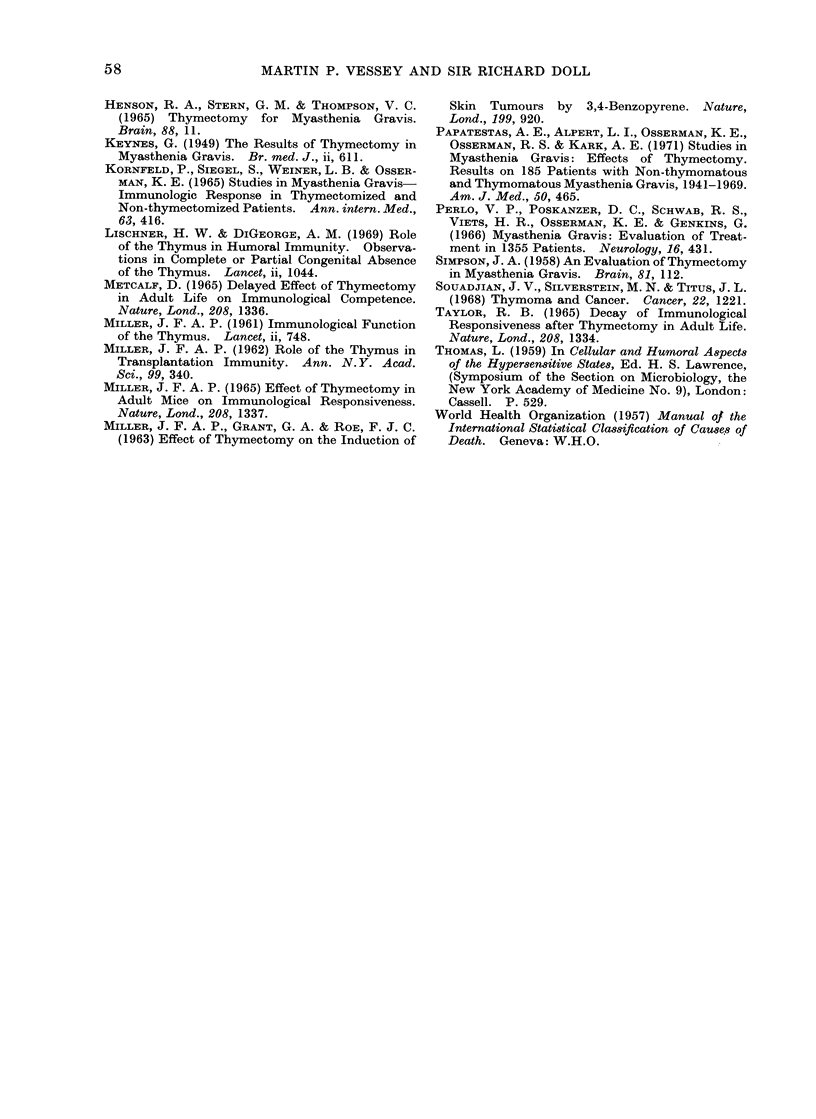# Thymectomy and Cancer—A Follow-Up Study

**DOI:** 10.1038/bjc.1972.9

**Published:** 1972-02

**Authors:** Martin P. Vessey, Richard Doll

## Abstract

Three hundred and eighty-two patients undergoing thymectomy for myasthenia gravis at 4 London hospitals during the years 1942-64 have been followed to the end of 1967. Five of these patients died from extrathymic tumours while 5·5 would have been expected to do so from the national experience. An additional 5 patients developed non-fatal extrathymic tumours during the period of follow-up. These data provide no evidence that adult thymectomy is followed by an increased risk of neoplastic disease, but more prolonged follow-up will be required before a final conclusion can be drawn.


Br. J. Cancer (1972) 26, 53

### THYMECTOMY AND CANCER—A FOLLOW-UP STUDY

MARTIN P. VESSEY AND SIR RICHARD DOLL

*Regius Department of Medicine, Radcliffe Infirmary, Oxford*

Received for publication November 1971

**Summary.—Three hundred and eighty-two patients undergoing thymectomy for myasthenia gravis at 4 London hospitals during the years 1942–64 have been followed to the end of 1967. Five of these patients died from extrathymic tumours while 5·5 would have been expected to do so from the national experience. An additional 5 patients developed non-fatal extrathymic tumours during the period of follow-up. These data provide no evidence that adult thymectomy is followed by an increased risk of neoplastic disease, but more prolonged follow-up will be required before a final conclusion can be drawn.**

In 1959, Thomas suggested that the phenomenon of homograft rejection might represent a mechanism for natural defence against neoplastic disease. By this he implied that there was an immunological system for recognizing and eliminating malignant cells arising within the body, a concept that was developed by Burnet (1970) and characterized as "immunological surveillance".

If the body does possess natural immunological defence mechanisms against cancer, there is good reason to believe that the thymus will play an important part in them. In 1961, Miller first showed that thymectomy of newborn mice results in depletion of the lymphocyte population of the blood and lymphoid tissues, an inability to reject foreign skin grafts, and premature death attributed to infection. Since that time, it has been shown in a number of species that neonatal thymectomy impairs immune reactions mediated by small lymphocytes, such as transplantation immune reactions (Miller, 1962) and delayed sensitivity reactions (Arnason *et al.*, 1962). The humoral antibody responses to most antigens are not affected and immunoglobulin production is not impaired (Fahey *et al.*, 1965). The thymus is thus concerned with the type of reaction which may be of importance in "immunological surveillance".

Thymectomy in adult rodents was originally thought to be without any important effect, but it was subsequently shown to be followed by a decline in immunological capacity which becomes apparent only after a period of 6–9 months (Miller, 1965; Metcalf, 1965; Taylor, 1965). From this finding it was concluded that the adult thymus influences the development of a population of immunologically competent cells which are long lived, and that only when this pool has been depleted do defects in immune capacity become apparent (Miller, 1965).

A number of studies have indicated that thymectomy renders mice more susceptible to the carcinogenic activity of oncogenic viruses and certain chemical carcinogens. For example, Miller *et al.* (1963) found increased activity of a carcinogenic hydrocarbon in producing skin cancer in mice thymectomized at 3 days of age when compared with sham operated controls, while Gaugus *et al.* (1969) reported that 100% of mice thymectomized at 6 weeks of age and treated with antilymphocytic serum developed tumours of various types, thought to be due to polyoma virus. Tumours induced by oncogenic viruses and chemical carcinogens have been shown to possess distinct cellular antigens which can evoke a homograft type of reaction. The growth of these antigenic tumours is thus facilitated by inadequacy of transplantation immune reactions.

Information concerning the role of the thymus in man in adult life is sparse. Absence of the thymus is associated with gross defects of the immunological system (Lischner and DiGeorge, 1969), but infants with this defect have not survived sufficiently long to show what effect this might have in adult life. In adults, only patients with myasthenia gravis (with or without thymoma) have been subjected to thymectomy in any number and studies of the immunological status of such patients are both few and confusing. For example, Kornfeld *et al.* (1965) found a normal primary response to H-agglutinin after triple typhoid vaccination in both thymectomized and non-thymectomized myasthenics, but found evidence of an impaired secondary response after later challenge. The impairment was greater in thymectomized than in non-thymectomized patients, but the authors were uncertain whether removal of the thymus or the greater severity of the disease in patients who had come to operation was responsible for the difference. They were unable to find any impairment of established delayed hypersensitivity to a variety of antigens, or induced hypersensitivity to dinitrochlorobenzene. Adner *et al.* (1964), on the other hand, found a diminished capacity among their myasthenic patients, both thymectomized and non-thymectomized, to acquire dinitrochlorobenzene sensitivity.

Although a number of follow-up studies of patients subjected to thymectomy for myasthenia gravis have been reported (see discussion), they have been concerned principally with the response of the disease to the operation rather than with the cancer risk among the survivors. Accordingly, we thought it would be worthwhile identifying a large group of thymectomized myasthenics and following them up to find out how many subsequently died from extrathymic malignant disease. The results of this investigation form the subject of the present report.

### MATERIAL AND METHODS

A total of 423 patients who underwent thymectomy for myasthenia gravis between January 1, 1942, and December 31, 1964, at 4 London hospitals (the National Hospital for Nervous Diseases, New End Hospital, St Bartholomew's Hospital and the London Hospital) were identified from diagnostic indexes and operating theatre books. Four patients could not be studied further because their case records had been lost, but every effort was made to follow the remaining 419 to December 31, 1967. Death certificates were obtained for those who had died, and the causes of death were classified according to the Seventh Revision of the International Classification of Diseases (World Health Organization, 1957). Special enquiries were made about all patients certified as dying from an extrathymic tumour and information was also sought about the occurrence of non-fatal tumours by searching all available case records and by corresponding with general practitioners.

Thirty-six of the 419 patients were foreign nationals who had come to Britain for the operation. These patients are not considered further here, but we may note that none of the 29 who were successfully followed to December 31, 1967, had, so far as we could ascertain, developed a fatal or a non-fatal extrathymic tumour.

All but one of the remaining 383 patients were successfully followed to December 31 1967. Some of the characteristics of these patients are shown in Table I. More than two-thirds were female, and about one-sixth had a thymoma. Patients with a thymoma were older on average at the time of operation than those without.

The numbers of years that these patients had been at risk of dying were computed separately for those with and without thymoma, for each sex and 5-year age group, for each year after thymectomy, and for each calendar year of observation. These numbers were then multiplied by the corresponding death rates for England and Wales and an

TABLE I.—*Distribution of the 382 Patients Included in the Analysis According to Sex, Thymic Pathology and Age at Thymectomy (percentages in parentheses)*

| Sex | Thymic pathology | Age at thymectomy (years): Up to 19 | 20–39 | 40 or more | Total |
|---|---|---|---|---|---|
| M | No thymoma | 14 (*16·5*) | 47 (*55·3*) | 24 (*28·2*) | 85 |
| M | Thymoma | 0 (—) | 7 (*30·5*) | 16 (*69·5*) | 23 |
| F | No thymoma | 50 (*21·5*) | 136 (*58·6*) | 46 (*19·9*) | 232 |
| F | Thymoma | 0 (—) | 11 (*26·2*) | 31 (*73·8*) | 42 |

TABLE II.—*Observed and Expected Numbers of Deaths by Cause According to Sex and Thymic Pathology*

| | | Cause of death | | | | | |
|---|---|---|---|---|---|---|---|
| | | Myasthenia or thymoma | | Extrathymic tumours | | All other causes | |
| Sex | Thymic pathology | Obs. | Exp. | Obs. | Exp. | Obs. | Exp. |
| M | No thymoma | 15 | 0* | 0 | 1·96 | 7 | 6·31 |
| M | Thymoma | 14 | 0* | 0 | 0·42 | 2 | 1·20 |
| F | No thymoma | 47 | 0* | 5 | 2·60 | 4 | 4·84 |
| F | Thymoma | 24 | 0* | 0 | 0·54 | 2 | 1·22 |
| Total | | 100 | 0* | 5 | 5·52 | 15 | 13·57 |

* Not calculated, but approximates closely to zero.

estimate obtained of the numbers of deaths that would have been expected if the patients had suffered the same mortality as the general population.

### RESULTS

Table II shows the observed and expected numbers of deaths among the 382 patients up to December 31, 1967, (i) from myasthenia gravis or thymoma, (ii) from extrathymic tumours, and (iii) from all other causes of death, classified by the sex of the patient and the thymic pathology. The only major discrepancy between the observed and the expected numbers of deaths is the anticipated one relating to myasthenia gravis and thymoma. So far as extrathymic tumours are concerned, the total of 5 observed is almost the same as the 5·5 expected.

Table III shows how the 5 deaths from extrathymic tumours were distributed with respect to age and interval since thymectomy. The most notable feature is that 3 fatalities were observed at ages 20–39 years while only 0·51 would have been expected; but with such small numbers little weight can be attached to this finding.

Information concerning the nature of the 5 fatal tumours is given in Table IV, which also provides details of 5 non-fatal tumours diagnosed during the follow-up period. There is no suggestion from these data that any particular type of tumour tends to develop after thymectomy.

The full analysis of the present data relates only to the period up to December 31, 1967, but during our enquiries we obtained some additional information about the progress and occurrence of tumours in later years. First, the patient listed as case 7 in Table IV died in May 1968; no post-mortem was held and the site of the primary tumour was never found. Biopsy of secondary deposits during life showed the tumour to be poorly differentiated with a glandular structure in some areas. Secondly, a male patient died from carcinoma of the stomach in July 1969 at the age of 59 years, 13 years after a thymoma had been removed. Thirdly, a female patient developed carcinoma of the breast in February 1968 at the age of 53 years, 21 years after

TABLE III.—*Numbers of Deaths from Extrathymic Tumours Observed and Expected by Age Groups and Interval Since Thymectomy. Patients of Both Sexes With and Without Thymoma*

| | Interval since thymectomy (years) | | | | | | | | | |
|---|---|---|---|---|---|---|---|---|---|---|
| | Up to 4 | | 5–9 | | 10–14 | | 15 or more | | Total | |
| Age( years) | Obs. | Exp. | Obs. | Exp. | Obs. | Exp. | Obs. | Exp. | Obs. | Exp. |
| Up to 19 | 0 | 0·01 | 0 | 0·00 | 0 | 0·00 | 0 | 0·00 | 0 | 0·01 |
| 20–39 | 0 | 0·18 | 1 | 0·17 | 2 | 0·11 | 0 | 0·05 | 3 | 0·51 |
| 40–59 | 0 | 0·81 | 0 | 0·87 | 2 | 0·91 | 0 | 0·85 | 2 | 3·44 |
| 60 or more | 0 | 0·22 | 0 | 0·27 | 0 | 0·47 | 0 | 0·60 | 0 | 1·56 |
| Total | 0 | 1·22 | 1 | 1·31 | 4 | 1·49 | 0 | 1·50 | 5 | 5·52 |

TABLE IV.—*Extrathymic Tumours Occurring in the* 382 *Patients Included in the Analysis up to December* 31, 1967

| Case number | Sex | Thymic pathology | Age at diagnosis (years) | Age at death (years) | Interval since thymectomy at diagnosis (years) | Nature of tumour |
|---|---|---|---|---|---|---|
| Fatal tumours | | | | | | |
| 1 | F | No thymoma | 28 | 29 | 8 | Hodgkins disease |
| 2 | F | No thymoma | 34 | 34 | 13 | Astrocytoma of spinal cord |
| 3 | F | No thymoma | 36 | 37 | 10 | Osteogenic sarcoma of sacrum |
| 4 | F | No thymoma | 42 | 45 | 8 | Carcinoma of breast |
| 5 | F | No thymoma | 52 | 53 | 13 | Squamous carcinoma of abd. wall* |
| Non-fatal tumours | | | | | | |
| 6 | M | Thymoma | 43 | — | 11 | Carcinoma of breast |
| 7 | M | No thymoma | 45 | — | 11 | Metastatic carcinoma of spine† |
| 8 | F | No thymoma | 53 | — | 16 | Chronic lymphatic leukaemia |
| 9 | F | Thymoma | 55 | — | 13 | Bronchial adenoma-carcinoid type |
| 10 | F | No thymoma | 57 | — | 16 | Pituitary adenoma |

* Complicating faecal fistulae thought to be secondary to tuberculous peritonitis in early life.
† Primary never found.

thymectomy (no thymoma found). Finally, a rodent ulcer of the nose was diagnosed in a 60-year-old female patient in January 1969, 26 years after thymectomy (no thymoma found).

### DISCUSSION

So far as they go, the results of the present study do not suggest that thymectomy in adult life leads to any special risk of neoplastic disease either as a whole or of any particular type. It should be noted, however, (i) that observations extending beyond 15 years after operation are few at present, (ii) that 3 of the deaths from neoplastic disease which occurred before December 31, 1967, were in patients under 40 years of age, and (iii) that all 13 tumours that have been observed occurred 8 or more years after thymectomy. More prolonged follow-up of the present series of patients is therefore desirable.

A number of papers evaluating thymectomy as a treatment for myasthenia gravis have been published, concerning both patients operated on in Britain (*e.g.* Keynes, 1949; Simpson, 1958; Henson *et al.*, 1965) and patients operated on in the United States (*e.g.* Eaton and Clagett, 1950, 1955; Perlo *et al.*, 1966; Papatestas *et al.*, 1971). These publications are of little help in connection with the present problem, because none of them includes any special analysis of extrathymic neoplasms, and deaths are for the most part classified only as myasthenic or non-myasthenic. Furthermore, it should be noted that the major British papers mostly relate to patients included in the present series (Keynes, 1949; Simpson, 1958; Henson *et al.*, 1965). In none of the publications, however, has it been suggested that an undue number of patients developed cancer either among those subjected to thymectomy or among those who were treated medically.

Special mention must be made of a report by Souadjian *et al.* (1968). From 1925 to 1964, 213 patients seen at the Mayo Clinic had a histologically proved diagnosis of thymoma. Souadjian and his colleagues obtained follow-up information for 195 of these and studied 146 who had died and had an autopsy. How many of these patients also had myasthenia gravis is not stated, but presumably all of them had a thymectomy, although this also is not specified. Thirty-one of these 146 patients developed an extrathymic malignant lesion. The highest incidence of these extrathymic malignant lesions was 10–15 years after the diagnosis of a thymoma. Five of the 31 patients developed a lymphoma; the remaining 26 tumours affected a wide variety of organs. For comparative purposes Souadjian and his colleagues studied 177 patients with parathyroid adenoma; only 15 of these patients subsequently developed a malignant neoplasm of another tissue despite their more favourable survival experience.

These findings of Souadjian and his colleagues are difficult to interpret. Patients who had not died were specifically excluded, and the analysis was not conducted on a formal actuarial or person-years at risk basis. The only information relevant to the latter point is that 81 of the 146 patients with a thymoma survived more than 5 years after diagnosis. None the less, the number of patients developing extrathymic malignancies seems remarkably high. Our findings do not support those of Souadjian and his colleagues, but it should be noted that among the 65 patients with a thymoma included in the present analysis, less than 100 person-years at risk 10 or more years after thymectomy had accumulated by December 31, 1967. One possibility that might be considered is that the thymectomies in our patients were incomplete. This, however, is not borne out by the autopsy evidence. Of the 120 patients who died (Table II), we were able to examine the autopsy reports for 43. Five of these 43 died from extension of a malignant thymoma, but in none of the remaining 38 was any residual thymic tissue noted at post-mortem.

We would like to express our gratitude to Mrs B. Norman-Smith who was responsible for tracing the patients; to Miss E. J. Armour, Dr J. N. Blau, Mr G. F. Flavell, Dr R. A. Henson, Mr G. K. Horner, Mr M. J. Lange, Professor J. A. Simpson, Dr G. Stern and Mr V. C. Thompson, all of whom assisted in identifying patients who had had a thymectomy; to physicians and surgeons at the National Hospital, New End Hospital, St Bartholomew's Hospital, and the London Hospital, for permission to study patients under their care; and to Mr I. D. Hill for help with the analysis.

### REFERENCES

Adner, M. M., Sherman, J. D., Ise, C., Schwab, R. & Dameshek, W. (1964) Immunologic Survey of Patients with Myasthenia Gravis. *New Engl. J. Med.*, *271*, 1327.

Arnason, B. G., Jankovic, B. D., Waksman, B. H. & Wennerstein, C. (1962) Role of the Thymus in Immune Reactions in Rats. II. Suppressive Effect of Thymectomy at Birth on Reactions of Delayed (Cellular) Hypersensitivity and the Circulating Small Lymphocyte. *J. exp. Med.*, *116*, 177.

Burnet, M. (1970) *Immunological Surveillance*. London: Pergamon Press.

Eaton, L. M. & Clagett, O. T. (1950) Thymectomy in the Treatment of Myasthenia Gravis. Results in 72 Cases Compared with 142 Control Cases. *J. Am. med. Ass.*, *142*, 963.

Eaton, L. M. & Clagett, O. T. (1955) Present Status of Thymectomy in Treatment of Myasthenia Gravis. *Am. J. Med.*, *19*, 703.

Fahey, J. L., Barth, W. F. & Law, L. W. (1965) Normal Immunoglobulins and Antibody Response in Neonatally Thymectomized Mice. *J. natn. Cancer Inst.*, *35*, 663.

Gaugas, J. M., Chesterman, F. C., Hirsch, M. S., Rees, R. J. W., Harvey, J. J. & Gilchrist, L. (1969) Unexpected High Incidence of Tumours in Thymectomized Mice Treated with Anti-lymphocytic Globulin and *Mycobacterium leprae*. *Nature, Lond.*, *221*, 1033.

HENSON, R. A., STERN, G. M. & THOMPSON, V. C. (1965) Thymectomy for Myasthenia Gravis. *Brain*, *88*, 11.

KEYNES, G. (1949) The Results of Thymectomy in Myasthenia Gravis. *Br. med. J.*, ii, 611.

KORNFELD, P., SIEGEL, S., WEINER, L. B. & OSSERMAN, K. E. (1965) Studies in Myasthenia Gravis—Immunologic Response in Thymectomized and Non-thymectomized Patients. *Ann. intern. Med.*, *63*, 416.

LISCHNER, H. W. & DIGEORGE, A. M. (1969) Role of the Thymus in Humoral Immunity. Observations in Complete or Partial Congenital Absence of the Thymus. *Lancet*, ii, 1044.

METCALF, D. (1965) Delayed Effect of Thymectomy in Adult Life on Immunological Competence. *Nature, Lond.*, *208*, 1336.

MILLER, J. F. A. P. (1961) Immunological Function of the Thymus. *Lancet*, ii, 748.

MILLER, J. F. A. P. (1962) Role of the Thymus in Transplantation Immunity. *Ann. N.Y. Acad. Sci.*, *99*, 340.

MILLER, J. F. A. P. (1965) Effect of Thymectomy in Adult Mice on Immunological Responsiveness. *Nature, Lond.*, *208*, 1337.

MILLER, J. F. A. P., GRANT, G. A. & ROE, F. J. C. (1963) Effect of Thymectomy on the Induction of Skin Tumours by 3,4-Benzopyrene. *Nature, Lond.*, *199*, 920.

PAPATESTAS, A. E., ALPERT, L. I., OSSERMAN, K. E., OSSERMAN, R. S. & KARK, A. E. (1971) Studies in Myasthenia Gravis: Effects of Thymectomy. Results on 185 Patients with Non-thymomatous and Thymomatous Myasthenia Gravis, 1941–1969. *Am. J. Med.*, *50*, 465.

PERLO, V. P., POSKANZER, D. C., SCHWAB, R. S., VIETS, H. R., OSSERMAN, K. E. & GENKINS, G. (1966) Myasthenia Gravis: Evaluation of Treatment in 1355 Patients. *Neurology*, *16*, 431.

SIMPSON, J. A. (1958) An Evaluation of Thymectomy in Myasthenia Gravis. *Brain*, *81*, 112.

SOUADJIAN, J. V., SILVERSTEIN, M. N. & TITUS, J. L. (1968) Thymoma and Cancer. *Cancer*, *22*, 1221.

TAYLOR, R. B. (1965) Decay of Immunological Responsiveness after Thymectomy in Adult Life. *Nature, Lond.*, *208*, 1334.

THOMAS, L. (1959) In *Cellular and Humoral Aspects of the Hypersensitive States*, Ed. H. S. Lawrence, (Symposium of the Section on Microbiology, the New York Academy of Medicine No. 9), London: Cassell. P. 529.

World Health Organization (1957) *Manual of the International Statistical Classification of Causes of Death*. Geneva: W.H.O.